# Role of Fractalkine-CX3CR1 Axis in Acute Rejection of Mouse Heart Allografts Subjected to Ischemia Reperfusion Injury

**DOI:** 10.3389/ti.2022.10157

**Published:** 2022-02-01

**Authors:** Taichi Kanzawa, Daisuke Tokita, Kan Saiga, Takafumi Yamakawa, Hidetoshi Ishigooka, Hironori Fukuda, Haruki Katsumata, Satoshi Miyairi, Rumi Ishii, Toshihito Hirai, Toshio Imai, Masayoshi Okumi, Kazunari Tanabe

**Affiliations:** ^1^ Department of Urology, Tokyo Women’s Medical University, Tokyo, Japan; ^2^ Clinical and Academic Research Promotion Center, Tokyo Women’s Medical University, Tokyo, Japan; ^3^ Department of Urology, Jyoban Hospital of Tokiwa Foundation, Fukushima, Japan; ^4^ KAN Research Institute Inc., Kobe, Japan

**Keywords:** transplantation, rejection, ischemia-reperfusion injury, fractalkine, CX3CR1, monocyte

## Abstract

Transplantation outcomes are affected by the increase in rejection associated with ischemia reperfusion injury (IRI). Fractalkine (FKN), a chemokine for recruitment of CX3CR1^+^ leukocytes, contributes to the pathogenesis of various inflammatory diseases. Herein, we evaluated the importance of the FKN-CX3CR1 axis during IRI-related rejections using a mouse heterotopic heart transplantation model. FKN expression and graft survival was compared between wild-type C57BL/6 recipients transplanted with BALB/c hearts preserved for 8 (WT-IRI) and 0.5 h (WT-control) at 4°C. Graft survival of WT-IRI was shorter than that of WT-control. FKN was expressed on the vascular endothelium in WT-IRI allografts, but minimally in WT-control. The role of the FKN-CX3CR1 axis in IRI-related rejection was directly investigated using the transplant model with CX3CR1-deficient recipients (CX3CR1 KO-IRI) or treatment with anti-mouse FKN monoclonal antibodies. Graft survival of CX3CR1 KO-IRI was longer than that of WT-IRI; antibody treatment prolonged graft survival. The contribution of CX3CR1^+^ monocytes to IRI-related rejection was evaluated by adoptive transfer to CX3CR1 KO-IRI. Adoptive transfer of CX3CR1^+^ monocytes attenuated the effect of prolonged graft survival in CX3CR1 KO-IRI. Overall, the FKN-CX3CR1 axis plays a major role during IRI-related rejection; its blockade has the potential to improve the outcomes of deceased donor transplantation.

## Graphical Abstract

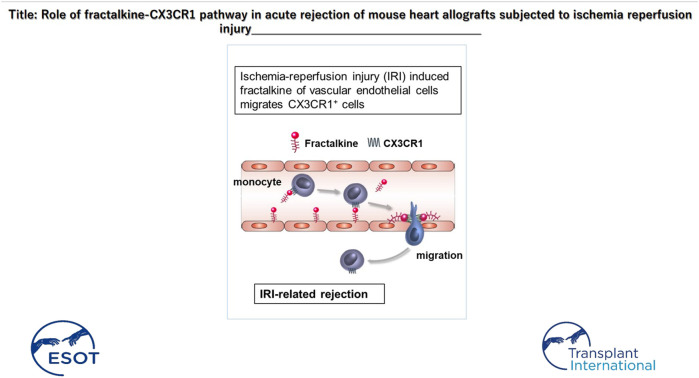

## Introduction

Generally, organs transplanted from living donors have superior function and survival compared with those from deceased donors. The longer the ischemic time imposed on grafts, the lower their function and patient survival rates, which are related to promotion of graft rejection in ischemia reperfusion injury (IRI) (1–8). Length of ischemic time is connected to the severity of primary graft dysfunction caused by the generation of oxygen radicals and activation of complement and endothelial cell dysfunction soon after the restoration of blood flow (9–11). During the early inflammatory process after reperfusion, the production of proinflammatory cytokines, including tumor necrosis factor (TNF)-α, interleukin (IL)-1, and IL-6, is greatly increased, and these cytokines induce and enhance alloimmune responses with the expression of adhesion molecules, such as intercellular adhesion molecule‐1 (ICAM‐1) and vascular cell adhesion molecule‐1 (VCAM‐1), on vascular endothelium, leukocyte infiltration, and tissue injury in grafts (11–14). Increased graft-infiltrating neutrophils, monocytes, and memory CD8^+^ T cells have been observed in donor organs subjected to prolonged cold ischemia, and these immune cells are implicated in graft dysfunction and transplant rejection (2, 15). Therefore, control of inflammatory cell infiltration in the initial process could be an effective approach to improve graft survival in allogeneic transplantation with donor organs subjected to prolonged ischemic conditions.

Fractalkine (FKN) is the only CX3C chemokine reported to date (16, 17). FKN expression is induced by stimulation of proinflammatory cytokines, such as TNF-α, IL-1, and interferon (IFN)-γ, and the translation product is expressed as a membrane-bound form on vascular endothelial cells (16). Membrane-bound FKN is cleaved by metalloproteinases, including TNF-α cleavage enzyme (TACE) and ADAM10, and released into the blood (18, 19). Soluble FKN acts as a chemokine that migrates immune cells expressing the FKN receptor, CX3CR1 (20), through integrin-independent and -dependent mechanisms (21). CX3CR1-expressing cells include a variety of leukocytes, such as monocytes, macrophages, cytotoxic effector lymphocytes, and natural killer (NK) cells, which migrate along a gradient of soluble FKN and enter through membrane-bound FKN expressed on the endothelium at inflamed sites (16, 22). The FKN-CX3CR1 axis is thought to be involved in the initiation of the innate immune system as well as the continuation of acquired immune response, and is known to play a role in immune defense against infections and tumors (23–27).

In contrast, FKN reportedly contributes to the pathological process of vascular and tissue injury in inflammation-mediated diseases and pathological conditions, including atherosclerosis, glomerulonephritis, rheumatoid arthritis, and transplant rejection, by enhancing migration and adhesion of CX3CR1-expressing leukocytes and promoting their transmigration to inflammatory sites (24, 27, 28). In a mouse heart transplantation model, FKN expression was increased in rejecting grafts, and anti-CX3CR1 neutralizing antibody treatment substantially prolonged graft survival (29). Prophylactic or therapeutic administration of anti-FKN monoclonal antibodies to a mouse collagen-induced arthritis model suppressed the migration of osteoclast progenitor cells derived from a monocyte/macrophage lineage of bone marrow cells into the joint while markedly improving synovitis and joint destruction (30). Furthermore, antibody clone 5H8 reduced skin fibrosis in a systemic sclerosis model (31).

Fractalkine has been reported to exert an effect on monocytes. CD14^+^ monocytes express CX3CR1 (20), and FKN induces migration (16) and enhances integrin-dependent cell adhesion in monocytes (21,32,33). It has also been documented that CX3CR1 regulates the retention of inflammatory monocytes in blood vessels during inflammation (34). In addition, migration of inflammatory monocytes via a mechanism dependent on the FKN-CX3CR1 axis has been reported to play an important role in renal injury after ischemia reperfusion by cross-clamping kidney pedicles (35). The FKN-CX3CR1 axis is also associated with the patrolling behavior of CD115^+^Gr-1^low/-^ monocytes crawling over the venous endothelium of the inflamed colon in a colitis model, locally producing proinflammatory cytokines and chemokines that promote subsequent leukocyte activation and infiltration. Anti-FKN antibody rapidly eliminated these crawling monocytes and inhibited their patrolling behavior (36).

To date, there have been many reports concerning the FKN-CX3CR1 axis and monocytes in inflammation-mediated pathogenesis, but their relevance to IRI-induced enhancement of rejection that occurs from an early stage after transplantation (IRI-related rejection) has not yet been fully clarified. We here assumed that FKN expression would be induced in grafts under long-term ischemia conditions and that monocytes infiltrated via the FKN-CX3CR1 axis have a significant impact on promotion of transplant rejection and graft failure. In the present study, we investigated the role of the FKN/CX3CR1 axis in a mouse model of IRI-related rejection using CX3CR1-deficient mice as recipients or an intervention with anti-mouse FKN neutralizing antibody (anti-FKN mAb), the emphasis being on the contribution of CX3CR1-positive monocytes.

## Materials and Methods

### Animals

Male BALB/c and C57BL/6 mice were purchased from the Japan SLC Corporation (Hamamatsu, Japan). CX3CR1 homogenous knockout in C57BL/6 background mice was performed by KAN Research Institute (Kobe, Japan). All mice were bred and maintained under specific pathogen-free conditions at the Institute of Laboratory at Tokyo Women’s Medical University (Tokyo, Japan). The pathogen-free conditions implemented were based on the criteria of the Central Institute for Experimental Animals (Kawasaki, Japan). The Tokyo Women’s Medical University internal committee on the use and care of laboratory animals approved all experiments (Reference ID: AE19-081).

### Ectopic Heart Transplantation

All transplant procedures were performed under general anesthesia using sevoflurane. Fully vascularized ectopic heart grafts from BALB/c donors were transplanted into C57BL/6 or CX3CR1-deficient recipients using microsurgical techniques (37). To investigate the influence of IRI on allograft rejection, ectopic heart transplantation was performed after donor hearts were preserved at 4°C for 8 h (prolonged cold ischemia: IRI) or 0.5 h (minimal cold ischemia: non-IRI control) (15). Cytotoxic T lymphocyte antigen 4-immunoglobulin (CTLA4-Ig) (ORENCIA^®^, Bristol-Myers Squibb, Lawrenceville, NJ, United States ) was administered intraperitoneally at a dose of 0.25 mg/day on the day of transplantation (day 0) and day 1. Graft engraftment was assessed by palpation with the presence of contraction as an indicator. Rejection was defined as complete cessation of contraction.

### Immunofluorescence Analysis

After perfusion fixation with 1% paraformaldehyde, heart grafts were collected and embedded with O.C.T. compound (Sakura Finetek Japan Co., Ltd, Tokyo, Japan). The tissues were cut into 6 μm-thick sections, blocked with normal donkey serum, and stained with 10 μg/ml of goat anti-rat FKN antibody (R&D Systems, Inc., Minneapolis, MN, United States ) and 50-fold diluted rabbit anti-mouse CD31 antibody (Abcam Plc, Cambridge, UK). The combination of 500-fold diluted Alexa fluor 555-conjugated donkey anti-goat IgG (Abcam) and Alexa fluor 488-conjugated goat anti-rabbit IgG (Abcam) was employed as the set of secondary antibodies. Nuclei were stained with 4′,6-diamidino-2-phenylindole (DAPI).

### Pathological Analysis

Grafts were procured at day 7 and hematoxylin-eosin staining was performed after fixation with 10% neutral phosphate-buffered formalin.

### Administration of anti-FKN mAb

Anti-FKN mAb (clone 5H8) and control IgG (anti-dinitrophenol mAb) (30, 31, 36) were provided by KAN Research Institute. Anti-FKN mAb or control IgG was administered at 500 μg/head on days−1, 3, 7, 10, and 14.

### Isolation and Adoptive Transfusion of CX3CR1-Positive Monocytes

Bone marrows recovered from 10 week-old wild-type C57BL/6 mice were treated with ammonium chloride buffer for hemolysis, and cultured for 3 days at 37°C in RPMI 1640 medium supplemented with 50 ng/ml of recombinant mouse macrophage colony-stimulating factor (R and D Systems), 10% fetal bovine serum, 0.1 mM 4-(2-hydroxyethyl)-1-piperazineethanesulfonic acid (HEPES) (pH 7.2–7.5), 1× MEM non-essential amino acid solution, 1 mM sodium pyruvate, 1× penicillin-streptomycin, and 100 μM 2-mercaptoethanol. Monocytes were isolated from post-culture cells using the CD115 MicroBead Kit (mouse; Miltenyi Biotec B.V. & Co. KG, Bergisch Gladbach, Germany). The isolated monocytes were transferred into CX3CR1-deficient recipients at 3×10^6^/head on day−1.

To confirm the purity of CX3CR1-positive monocytes in the isolated cells, Fc blocking with anti-CD16/CD32 antibody (BD Bioscience, San Jose, CA, United States ) was followed by staining with phycoerythrin (PE)-conjugated mouse CX3CR1 antibody and fluorescein isothiocyanate (FITC)-conjugated CD115 (AFS98; Tonbo Biosciences, San Diego, CA, United States ). Anti-mouse CX3CR1 antibody (clone L2D11) was provided by KAN Research Institute. Flow cytometry analysis was carried out with a FACSCanto^™^ II (BD Biosciences) and FlowJo software (Tree Star, Ashland, OR, United States ).

### Statistical Analysis

Comparisons of graft survival were analyzed by the log-rank test using Prism seven software (GraphPad Software, La Jolla, CA, United States ); differences with *p* values <.05 were considered significant.

## Results

### Enhanced Acute Rejection and Increased Expression of FKN on Vascular Endothelial Cells in Allografts Subjected to Longer Cold Ischemia.

**FIGURE 1:**
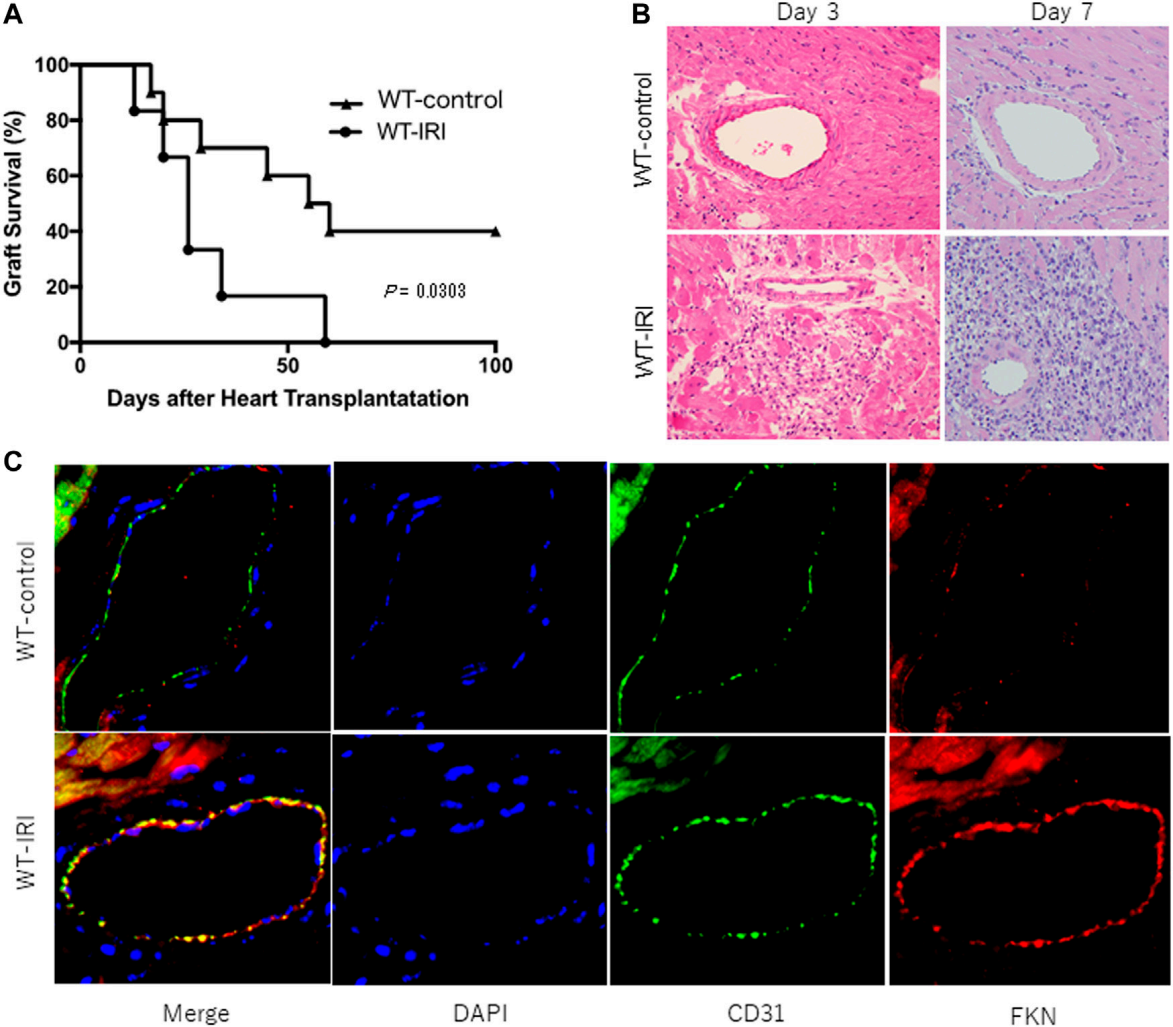
Reduced survival of mouse heart allografts owing to ischemia-reperfusion injury caused by prolonged cold ischemia. BALB/c hearts were transplanted into wild-type C57BL/6 recipients after 8 h of preservation at 4°C (WT-IRI: *n* = 6) or 0.5 h (WT-control: *n* = 10). CTLA4-Ig was administered intraperitoneally at a dose of 0.25 mg/day on the day of transplantation (day 0) and on day 1. **(A)** Graft survival. The comparison between groups was analyzed via the log-rank test. **(B)** Hematoxylin-eosin-stained images of graft tissues at day 3 and 7. **(C)** Expression of fractalkine (FKN) in graft tissues at day 3. Immunofluorescence staining was performed using anti-FKN (red) and anti-CD31 antibodies (green). Nuclei were stained with DAPI (blue).

To investigate the influence of IRI on allograft rejection, ectopic heart transplantation was performed on wild-type C57BL/6 recipients after donor hearts were preserved at 4°C for 8 h (WT-IRI) or 0.5 h (WT-control) and graft survival was compared between the two groups. As shown in Figure 1A, graft survival in the WT-IRI group was shorter than that in the WT-control group (median survival time (MST): WT-IRI = 26.0 days vs WT-control = 57.5 days; *p* = .0303, log-rank test). Histopathological findings at day 3 and 7 showed more severe cell infiltration in the WT-IRI group than in the WT-control group (Figure 1B). These results suggest that IRI was associated with enhanced graft rejection from the initial process (IRI-related rejection) and shortened graft survival in the WT-IRI group. To compare the expression of FKN in graft tissues between both groups, an immunofluorescence assay using anti-FKN antibody was performed on heart allografts at day 3. As shown in Figure 1C, FKN was strongly expressed on vascular endothelial cells in the WT-IRI group, which showed enhanced rejection and shortened graft survival compared to the WT control group, whereas less FKN signal was detected in the WT control group. In conclusion, the induction of FKN expression in vascular endothelial cells in graft tissues was dependent on IRI, which might affect IRI-related rejection and shortening graft survival.

### Lack of Influence of IRI on Allograft Survival in CX3CR1-Deficient Recipients

**FIGURE 2:**
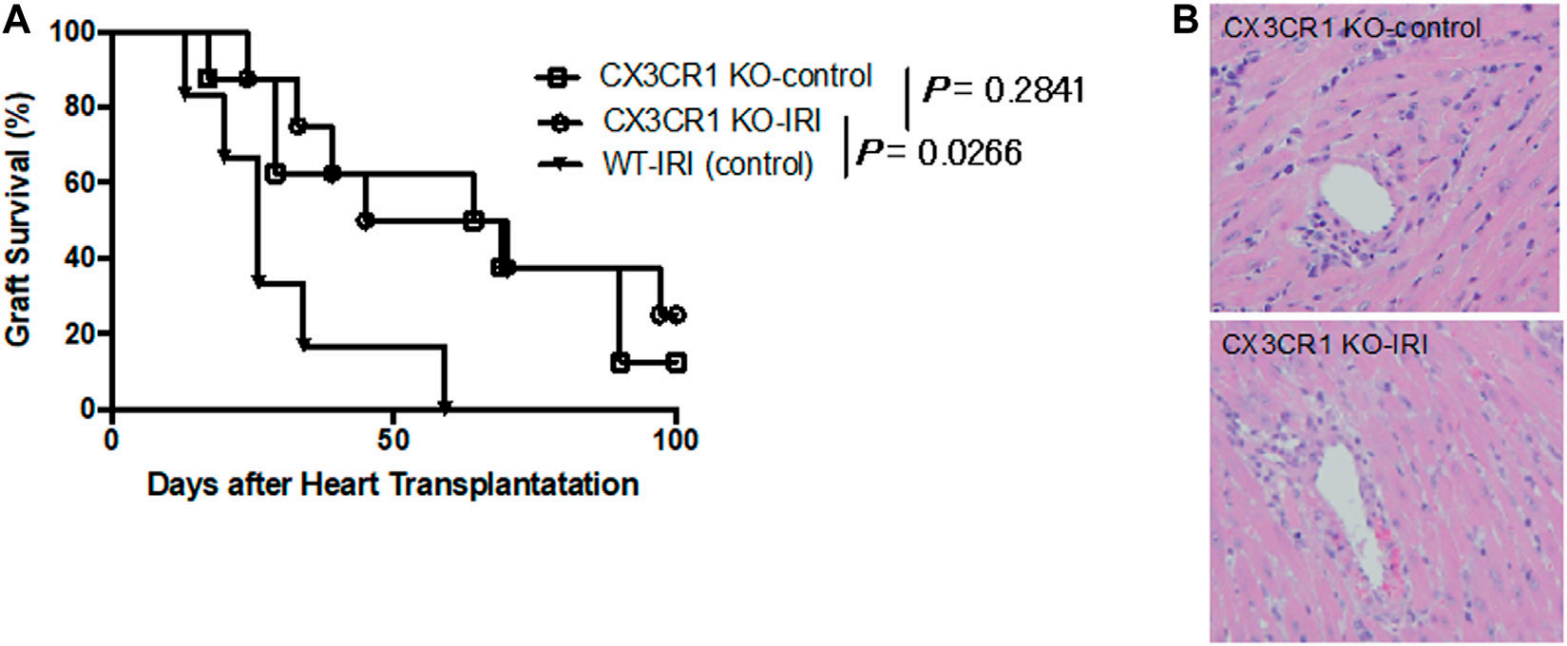
Loss of influence of ischemia-reperfusion injury on graft survival in CX3CR1-deficient recipients. CX3CR1-deficient mice (C57BL/6 background) were transplanted with BALB/c donor hearts preserved at 4°C for 8 h (CX3CR1 KO-IRI: *n* = 8) or 0.5 h (CX3CR1 KO-control: *n* = 8). CTLA4-Ig was administered intraperitoneally at a dose of 0.25 mg/day on the day of transplantation (day 0) and day 1. **(A)** Graft survival. The CX3CR1 KO-IRI group was compared with the CX3CR1 KO-control group or the WT-IRI group (see Figure 1). The comparisons were analyzed *via* the log-rank test.**(B)** Hematoxylin-eosin-stained images of graft tissues at day 7.

To investigate the relevance of the FKN receptor, CX3CR1, with respect to augmentation of acute rejection associated with cold ischemia, we transplanted BALB/c donor hearts preserved at 4°C for 8 h (CX3CR1 KO-IRI) or 0.5 h (CX3CR1 KO-control) into CX3CR1-deficient recipients. As shown in Figure 2A, there is no significant difference in graft survival between the CX3CR1 KO-IRI group (MST: 57.5 days) and the CX3CR1 KO-control group (MST: 66.5 days). Inflammatory cell infiltration in grafts at day 7 in both groups was similarly mild (Figure 2B). Furthermore, graft survival of the CX3CR1 KO-IRI group was significantly longer than that of the WT-IRI group (*p* = .0226, log-rank test) (Figure 2A). These results suggest that the presence of CX3CR1 molecules in recipients would be essential for IRI-related rejection.

### Improved Survival of Allografts Subjected to IRI in Wild-Type Recipients Receiving Anti-FKN mAb

**FIGURE 3:**
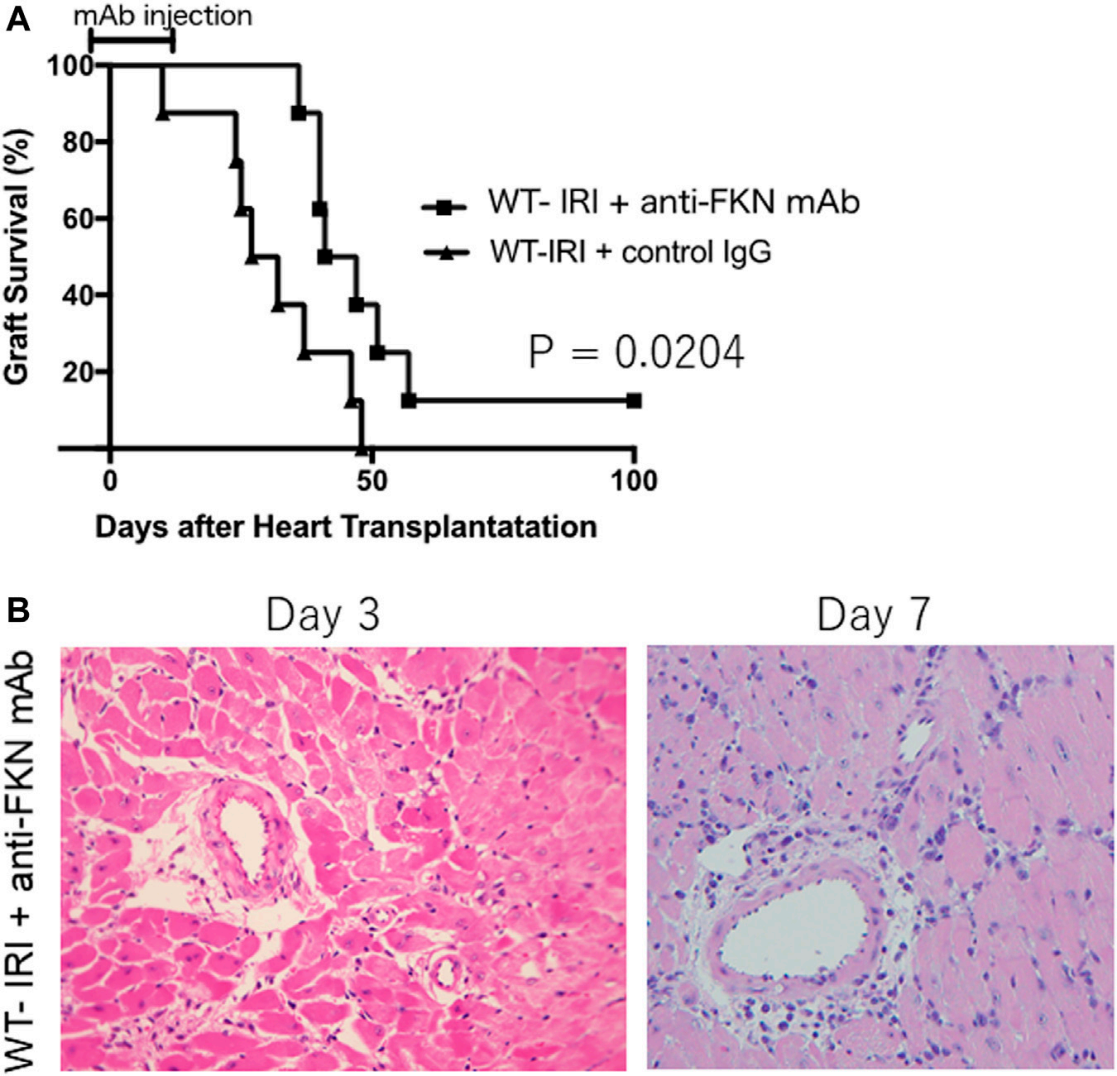
Preventive effect of anti-fractalkine monoclonal antibody (anti-FKN mAb) treatment on reduced allograft survival caused by cold ischemia. We transplanted BALB/c hearts preserved for 8 h at 4°C into wild-type C57BL/6 mice and treated with anti-FKN mAb (WT-IRI + anti-FKN mAb: *n* = 8) or control IgG (WT-IRI + control IgG: *n* = 8). Anti-FKN mAb clone 5 H8 was administered at 500 μg on the day before transplantation (day−1) and on days 3, 7, 10, and 14. CTLA4-Ig was administered intraperitoneally at a dose of 0.25 mg/day on days 0 and 1. **(A)** Graft survival. The comparison between groups was analyzed via the log-rank test. **(B)** A hematoxylin-eosin-stained image of graft tissues at day 3 and 7 in the WT-IRI + anti-FKN mAb group.

To further investigate the association between the FKN-CX3CR1 axis and IRI-related augmentation of rejection, we compared the survival of heart grafts subjected to cold ischemia in wild-type recipients with anti-FKN mAb treatment (WT-IRI + anti-FKN mAb) to those with control IgG treatment (WT-IRI + control IgG). As shown in Figure 3A, the WT-IRI + anti-FKN mAb group had a longer survival than the WT-IRI + control IgG group (MST: WT-IRI + anti-FKN mAb = 40.5 days vs WT-IRI + control IgG = 29.5 days; *p* = .0204, log-rank test). This result indicates that anti-FKN mAb treatment improves graft survival shortened by cold-ischemia conditions, confirming the importance of the FKN-CX3CR1 axis in IRI-related rejection. We compared the graft tissues at day 3 and 7 between the WT-IRI + anti-FKN mAb group (Figure 3B) and the WT-IRI group (Figure 1B) and found that the administration of anti-FKN mAb resulted in milder cellular infiltration (Figure 3B). These results indicate that blockade of the FKN-CX3CR1 axis inhibits IRI-related rejection.

### Importance of CX3CR1-Positive Monocytes in IRI-Related Rejection

**FIGURE 4:**
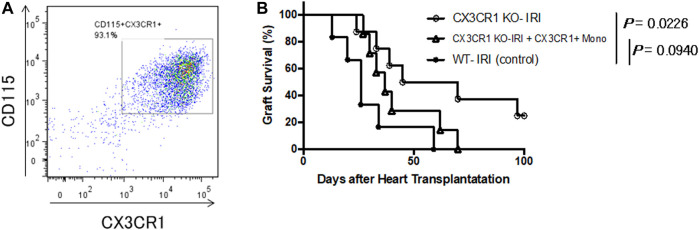
Restoration of the negative influence of cold ischemia on graft survival in CX3CR1-deficient recipients with adoptive transfer of CX3CR1-positive monocytes. Monocytes were purified from wild-type C57BL/6 bone marrow cells stimulated with macrophage colony-stimulating factor using magnetic beads conjugated with anti-CD115 monoclonal antibody. Approximately 3 × 10^6^ of the isolated monocytes were transferred to CX3CR1-deficient mice and the next day, BALB/c hearts preserved at 4°C for 8 h were transplanted (CX3CR1 KO-IRI + CX3CR1^+^ Mono: *n* = 7). CTLA4-Ig was administered intraperitoneally at a dose of 0.25 mg/day on the day of transplantation (day 0) and day 1. **(A)** Flow cytometric analysis of the isolated monocytes using anti-CD115 and anti-CX3CR1 antibodies. **(B)** The comparison of graft survival between the CX3CR1 KO-IRI + CX3CR1^+^ Mono group and the CX3CR1 KO-IRI (see Figure 3) or the WT-IRI group (see Figure 1) was analyzed via the log-rank test.

We focused on CX3CR1-positive monocytes as effector cells essential for enhancing acute rejection of grafts subjected to prolonged cold ischemia. C57BL/6 bone marrow cells were cultured under M-CSF stimulation for 3 days, and monocytes were purified with anti-CD115 mAb. Flow cytometry analysis showed that more than 93% of the isolated cells were CX3CR1-positive monocytes (Figure 4A). Approximately 3 × 10^6^ of the isolated cells were transferred into CX3CR1-deficient mice. The next day, donor hearts preserved for 8 h at 4°C were transplanted into the mice (CX3CR1 KO-IRI + CX3CR1^+^ Mono). As shown in Figure 4B, graft survival in the CX3CR1 KO-IRI group was significantly prolonged as compared to that in the WT-IRI group; adoptive transfer of CX3CR1-positive monocytes attenuated the effect of prolongation of graft survival. As a result of CX3CR1-positive monocyte infusion, there was no significant difference in graft survival between the CX3CR1 KO-IRI + CX3CR1^+^ Mono group (MST: 37.0 days) and the WT-IRI group (MST: 26.0 days). These results indicate that CX3CR1-positive monocytes are important effector cells in IRI-related rejection.

## Discussion

Prevention and mitigation of the impact of IRI is critical for protection against graft dysfunction and improvement of graft survival if the grafts have been ischemic for a long-time following recovery from donors. For this purpose, regulation of immune cell infiltration in grafts during the initial process would be highly effective. The FKN-CX3CR1 axis plays an important role in immune defense by controlling the migration and adhesion of various types of immune cells involved in immune responses at inflammatory sites or infected areas. Furthermore, the FKN-CX3CR1 axis has been reported to be involved in the development of inflammation-associated diseases and pathological conditions. Using a murine ectopic transplantation model with hearts subjected to cold ischemia, the present study clarified the importance of the FKN-CX3CR1 axis and CX3CR1-positive monocytes in IRI-related rejection, and then demonstrated the potential of the FKN-CX3CR1 axis as a target for prevention of post-transplant graft dysfunction and rejection of long-term preserved grafts, such as those recovered from deceased donors.

In the present study, we first analyzed the influences of cold ischemic time on grafts in a mouse heart transplantation model. The comparison between the grafts preserved for 8 h at 4 °C (IRI) and 0.5 h (non IRI; control) showed that graft survival in the WT-IRI group was significantly shorter than that in the WT-control group (Figure 1A), and more severe cell infiltration was observed in the graft pathology of the WT-IRI group as early as day 3 (Figure 1B). FKN expression on vascular endothelial cells was detected at day 3 in the WT-IRI group, but minimally in the WT-control group (Figure 1C). These results suggest that FKN expression on graft vascular endothelial cells increases infiltrating cells from an early stage and correlates with the severity of IRI-related rejection. It has been previously reported that the expression of FKN on endothelial cells was induced by proinflammatory cytokines, such as IL-1, IFN-γ, and TNF-α (16). Cold ischemia induced activation of the transcription factor, NF-κB, and consequently elevated expression of TNF-α in rat liver allografts (38). The increased levels of IL-1β, IL-6, and TNF-α in human renal graft vein plasma were observed during reperfusion after cold ischemia (39). From the previous findings and our own data here, we infer that in the WT-IRI group, the expression of FKN on endothelial cells would be facilitated by proinflammatory cytokines greatly induced in grafts subjected to IRI. Proinflammatory cytokines also regulate expression of various cell adhesion molecules (40, 41). Cold ischemia leads to expression of P-selectin and ICAM-1 on the endothelium, and augments allogeneic-mediated cell infiltration in rat kidney allografts (42). ICAM-1 antisense oligodesoxynucleotides prevent reperfusion injury and enhance immediate graft function during renal transplantation (13). The engagement of the FKN-CX3CR1 and integrin-ICAM-1 axes enhanced cell adhesion compared to each axis alone (24, 32, 43). Within the allografts subjected to cold ischemia in the WT-IRI group, cooperation of FKN with other molecules associated with cell adhesion would result in increasing infiltrating cells that correlates with the severity of IRI-related rejection.

Graft rejection in the present model is further complicated by influences of allogeneic immunity and IRI caused by cold ischemia. In this study, all experimental groups received intraperitoneal administration of CTLA4-Ig at a dose of 0.25 mg on the day of heart transplantation and the next day. CTLA4-Ig suppresses priming of alloimmune responses by inhibiting T-cell activation mediated by the CD28^−^CD80/CD86 co-stimulatory signals in antigen presentation (44–46) but reportedly has little in the way of suppressive effects on transplant rejection of allografts subjected to cold ischemia (15). In our preliminary study of recipients not receiving CTLA4-Ig, both grafts preserved for 8 h at 4°C and 0.5 h were rejected within 7 days and there was no difference in graft survival between them (data not shown). Acute rejection in these groups is thought to be predominantly caused by alloimmune responses owing to T-cell activation, which was independent of the influences of cold ischemia. The difference in graft survival between the WT-IRI and WT-control groups detected in the present study would be mainly based on IRI as they all survived over 7 days, the maximum survival when not receiving CTLA4-Ig that mitigates the effect of allogeneic immunity.

Next, to examine the importance of the FKN-CX3CR1 axis for graft rejection in cold-ischemic heart transplantation, we analyzed changes in graft survival employing CX3CR1-deficient mice as recipients or treatment with anti-FKN monoclonal antibodies. As shown in Figure 2A, in cases of transplantation with hearts subjected to cold ischemia for 8 h, CX3CR1-deficient recipients (CX3CR1 KO-IRI) exhibited prolonged graft survival compared to wild-type recipients (WT-IRI). As previously demonstrated, there is a significant difference in graft survival between donor hearts subjected to cold ischemia or not in wild-type recipients (Figure 1A: WT-IRI vs WT-control), but not in CX3CR1-deficient recipients (Figure 2A: CX3CR1 KO-IRI vs CX3CR1 KO-control). These results indicate that the loss of the FKN receptor, CX3CR1, almost completely abolishes the negative influence of cold ischemia on graft survival. Furthermore, when anti-FKN mAb was administered to the wild-type recipients transplanted with hearts subjected to prolonged cold ischemia (WT-IRI + anti-FKN mAb), a significant improvement in graft survival was observed (Figure 3). Taken together with these findings, the FKN-CX3CR1 axis plays a major role in IRI-related rejection.

Although we showed evidence herein for the relationships involving the FKN-CX3CR1 axis-mediated promotion of graft rejection owing to IRI, the importance of the FKN-CX3CR1 axis in allogeneic acute rejection has already been reported. During mouse heart transplantation, FKN expression was increased in the rejecting allografts and was prominent on vascular tissues and endothelium at early time points. Anti-FKN or anti-CX3CR1 antibodies inhibited the adhesion of peripheral blood mononuclear cells to the vascular endothelium, and treatment with anti-CX3CR1 antibody significantly prolonged survival of mouse cardiac allografts (29). Moreover, when CX3CR1 knockout mice were used as recipients, graft survival was prolonged in the presence of subtherapeutic levels of cyclosporin A with a concomitant reduction in infiltrating macrophages, NK cells, and other leukocytes observed in the grafts (28). These studies highlighted the importance of the FKN-CX3CR1 axis during the pathogenesis of acute transplant rejection. Based on these findings along with the present data, the FKN-CX3CR1 axis appears to be an effective target for not only preventing acute rejection but also controlling IRI-dependent promotion of rejection responses, suggesting that neutralizing antibodies or other blockers targeting the axis could contribute to protect the long-term preserved donor organs from graft failure.

Next, to evaluate the potential of CX3CR1-positive monocytes as immune cells that exert effector function during IRI-related rejection, CX3CR1-deficient recipients adoptively transferred with monocytes isolated from wild-type mice were transplanted with donor hearts subjected to prolonged cold ischemia (CX3CR1 KO-IRI + CX3CR1^+^ Mono). As shown in Figure 4B, no significant difference was found in graft survival between the CX3CR1 KO-IRI + CX3CR1^+^ Mono and WT-IRI groups. Moreover, CX3CR1-deficient recipients without transferring monocytes (CX3CR1 KO-IRI) exhibited prolonged survival compared to the WT-IRI group (Figure 1A). These results demonstrate that CX3CR1-positive monocytes play an important role in IRI-related rejection in the present model, suggesting that inhibition of monocyte migration through the FKN-CX3CR1 axis may have contributed to the improvement in graft survival shown using CX3CR1-deficient mice as recipients or by treatment with anti-FKN mAb. It has been reported that acute allograft dysfunction is closely related to monocyte infiltration and the monocyte/macrophage lineage cells function as effectors of allograft damage and activate allogeneic responses during acute allograft rejection (47–50). Monocytes that infiltrated into allografts through the FKN-CX3CR1 axis may have contributed to enhancement of graft rejection through similar mechanisms. Conversely, survival rates of the CX3CR1 KO-IRI + CX3CR1^+^ Mono group were slightly higher than those of the WT-IRI group (Figure 4B), suggesting that a transfer of CX3CR1-positive monocytes alone may not fully restore the influence of IRI on grafts exerted in the WT-IRI group. In addition to monocytes, CX3CR1 is known to be expressed in effector memory CD8^+^ T cells (51). Reportedly, there was a direct association between increased durations of cold ischemic allograft preservation and numbers/enhanced functions of early graft-infiltrating endogenous memory CD8^+^ T cells, which directly mediate rejection of allografts subjected to prolonged ischemia (15). Although the present data have demonstrated the crucial role of CX3CR1-positive monocytes in graft damage correlated with reduced survival of cold-preserved allografts, analysis of the involvement of other CX3CR1-positive cells is an important topic for future research.

Thus, although there is still room for analysis of immune cells correlated with enhancement of rejection owing to cold ischemia, the importance of the FKN-CX3CR1 axis was clearly demonstrated in the present study. These results strongly suggest that the FKN-CX3CR1 axis may be useful as an interventional target for prophylaxis and therapy to improve survival of allografts affected by IRI. As an initial attempt at clinical application of FKN-CX3CR1 blockades, a humanized mAb against FKN, E6011, has been evaluated in a clinical trial for rheumatoid arthritis. No serious adverse events or deaths were reported in this study, indicating that the FKN-CX3CR1 blockade intervention is safe and well-tolerated, and may have a positive clinical effect in patients with highly active rheumatoid arthritis (52, 53). Based on the present study, clinical applications of FKN-CX3CR1 blockades even in the field of transplantation would be expected, and anti-FKN antibodies hold great promise as an interventional approach to protect grafts that have been left in an ischemic state for a long-time during preservation, such as organs from deceased donors, and to improve the outcomes of organ transplantation.

There are potential limitations to the present study. The following test have not been performed: 1) to compare the early-stage histopathology of the CX3CR1 KO-control, CX3CR1 KO-IRI and WT-IRI groups, 2) to compare the donor-reactive memory CD8^+^ T cells of wild-type and CX3CR1-KO mice, and 3) to confirm the infiltration of transplanted monocytes into graft tissues and early-stage histopathology. These rigorous analyses are necessary to further validate the role of the FKN-CX3CR1 axis in IRI-related rejection in the future.

In conclusion, ischemia in donor organs over time with respect to transplantation leads to exacerbation of graft rejection via impaired reperfusion after transplantation. CX3CR1-positive monocytes and the FKN-CX3CR1 axis play important roles in this series of tissue disorders that significantly affect allograft survival. Blockade of the FKN-CX3CR1 axis by anti-FKN antibodies or other means reduces the impact of IRI-related rejection and could be an effective intervention to improve the outcomes of deceased donor transplantation.

## Capsule Sentence Summary

This study evaluated the significance of the FKN-CX3CR1 pathway during ischemia-reperfusion injury (IRI)-related graft rejections using a mouse heterotopic heart transplantation model. We believe that our study makes a significant contribution to understanding the mechanism because the roles of the FKN-CX3CR1 axis in IRI-related rejection were directly investigated using the transplant model with CX3CR1-deficient recipients (CX3CR1-KO IRI) or treatment with anti-mouse FKN monoclonal antibodies. Our findings indicated that the FKN-CX3CR1 axis plays a major part during IRI-related rejection and its blockade has the potential to improve the outcomes of deceased donor transplantation. Further, we believe that this paper will be of interest to the readership of your journal because it is known the outcome of transplantation is affected by the promotion of rejection associated with IRI. Our findings suggest there is a way to potentially mitigate this phenomenon and enhance the acceptance of graft transplantations from deceased donors.

## Data Availability

The original contributions presented in the study are included in the article/Supplementary Material, further inquiries can be directed to the corresponding authors.

## Abbreviations

CTLA4-Ig, cytotoxic T lymphocyte antigen 4-immunoglobulin; FITC, fluorescein isothiocyanate; FKN, fractalkine; IL, interleukin; HEPES, 4-(2-hydroxyethyl)-1-piperazineethanesulfonic acid; ICAM-1, intercellular adhesion molecule-1; IFN, interferon; IRI, ischemia reperfusion injury; mAb, monoclonal antibody; MST, median survival time; PE, phycoerythrin; TACE, TNFα cleavage enzyme; TNF, tumor necrosis factor; VCAM-1, vascular cell adhesion molecule-1.
